# Effects of novaluron exposure on cocooning and transcriptional changes of genes in silk gland of silkworm, *Bombyx mori*

**DOI:** 10.1038/s41598-026-50056-6

**Published:** 2026-04-26

**Authors:** Lin Zhu, Mengli Li, Yilin Ji, Mengjiao Wang, Guodong Zhao, Heying Qian

**Affiliations:** 1 Jiangsu Key Laboratory of Sericultural and Animal Biotechnology, School of Biotechnology, Jiangsu University of Science and Technology, Zhenjiang, 212100 China; 2 Key Laboratory of Silkworm and Mulberry Genetic Improvement, Ministry of Agriculture and Rural Affairs, Sericultural Scientific Research Center, Chinese Academy of Agricultural Sciences, Zhenjiang, 212100 China

**Keywords:** Novaluron, *Bombyx mori*, Silk gland, Cocooning, Gene expression, Developmental biology, Ecology, Ecology, Genetics, Molecular biology, Zoology

## Abstract

As a representative species of Lepidoptera, the silkworm (*Bombyx mori*) serves as both a model organism and an insect of economic importance. The abundant genetic mutation resources of silkworms provide a critical foundation for related studies. Globally, exposure to various pesticides often leads to failure in cocooning or direct mortality in silkworms. However, systematic investigations into the effects of novaluron exposure on silkworm growth, development, cocooning, and gene expressions are still lacking. This study revealed that novaluron exposure induced significant signs of intoxication in silkworms, accompanied by reductions in body weight and cocoon quality. Furthermore, novaluron exposure caused histopathological damage to the silk gland, reduced the cocooning rate, and significantly downregulated the expression of genes related to silk protein synthesis. Concurrent alterations were observed in the transcriptional levels of genes associated with the PI3K/Akt and CncC/Keap1 signaling pathways. In addition, novaluron exposure upregulated the relative expression levels of detoxification enzyme genes and enhanced corresponding enzymatic activities. These findings provided further insights into the response mechanisms of silkworms to novaluron exposure.

**Supplementary Information:**

The online version contains supplementary material available at 10.1038/s41598-026-50056-6.

## Introduction

*Bombyx mori*, is a major economic insect in China and many other countries and also serves as a model organism within the order Lepidoptera^1^. It classified under the family Bombycidae within Insecta, undergoes complete metamorphosis, progressing through four developmental stages (egg, larva, pupa, and moth) that are markedly distinct in both morphology and function^2^. These attributes, combined with its ease of maintenance, short life cycle, well-characterized genetic background, and manageable size for experiment, establish the silkworm as an ideal model system for genetic studies in insects^3^.

The silkworm’s diet consists almost exclusively of mulberry leaves^4^. Meanwhile, the ongoing intensification of agriculture has led to the widespread application of pesticides, which consequently has become a serious threat to silkworm survival and development^5^. The silkworm exhibits an extremely high sensitivity to most pesticides. Even trace amounts of pesticide residues can induce significant tissue damage, rendering it highly susceptible to adverse effects^6^. It has been documented that pyriproxyfen intoxication prevents silkworms from spinning silk and forming cocoons. In some cases, fifth-instar larvae display a false precocious maturity, which is characterized by developmental arrest and is frequently lethal as a consequence of impaired silk gland development^7^.

Novaluron, also known as flufenoxuron, is a benzoylurea insecticide belonging to the class of insect growth regulators (IGRs). Its application covers a wide range of crops including cotton, potatoes, citrus, corn, apples, and it also plays a significant role in the cultivation of solanaceous fruits and vegetables^8^. As a fluorinated IGR, novaluron exerts its insecticidal effect by potently inhibiting chitin synthesis, which disrupts the molting process of insects. This mechanism prevents the proper formation of a new exoskeleton, leading to lethal developmental malformations and ultimately death in target pests^9^. Studies have indicated that novaluron exposure can cause midgut damage in the silkworm, *Bombyx mori*, adversely affecting its physiological functions and development throughout the entire instars^10^. Furthermore, some researches demonstrated that novaluron not only impairs testicular tissue architecture and development, alters patterns of cell death, but also significantly reduces the egg-laying capacity of female moths^11^.

The silk gland serves as the primary organ responsible for the synthesis and secretion of silk proteins and can be anatomically and functionally divided into three distinct regions: the anterior, middle, and posterior silk glands^12^. Silk fibroin, the core structural protein, is composed of three major subunits: a heavy chain (Fib-H, 350 kDa), a light chain (Fib-L, 26 kDa), and P25 glycoprotein^13^. Meanwhile, sericin proteins function as water-soluble adhesive polymers that encapsulate and bind fibroin filaments together. These glue-like proteins, primarily encoded by genes such as *Ser-1*, *Ser-2*, and *Ser-3*, provide protective coating and structural cohesion to the silk fibers^14^. The coordinated interaction between fibroin and sericin within the silk gland enables the formation of a complete cocoon filament. This synergy imparts remarkable mechanical strength, toughness, and hygroscopic properties to the silk, allowing it to adapt to diverse and demanding environmental conditions.

Previous studies have demonstrated that the Forkhead box (Fox) gene family plays crucial regulatory roles in organ formation and development, in addition to participating in various biological processes such as metabolism and immune responses^15^. Silk Gland Factor 1 (SGF1), a member of the FoxA subfamily, was the first identified transcription factor involving in regulating silk protein synthesis in silkworm. It is essential for silk gland development, where it activates the expression of fibroin genes to promote fibroin production^16^. The activity of SGF1 is further modulated by the PI3K/Akt signaling pathway, which is known to be critical for the nutrient-dependent regulation of silk protein synthesis. The PI3K/Akt pathway is evolutionarily conserved across species including mice, fruit flies, and silkworms, and it regulates diverse physiological events such as cell proliferation, growth, and tissue differentiation^17,18^. One of its key downstream effectors, P70s6k, functions by phosphorylating the ribosomal protein S6, thereby modulating subsequent physiological activities^19^. Meanwhile, the CncC/Keap1 signaling pathway serves a vital function in metabolic detoxification and antioxidant responses. Importantly, research indicated that the PI3K/Akt and CncC/Keap1 pathways can coregulate the expressions of detoxification enzyme genes in response to insecticide-induced stress^20^.

Insect detoxification enzymes served as a group of heterogeneous enzymes with metabolic functions, capable of breaking down both endogenous and exogenous compounds^21^. This system primarily includes cytochrome P450 monooxygenases (CYPs), glutathione S-transferases (GSTs), and carboxylesterases (CarEs)^22^.

In this study, the toxic effects of novaluron on silkworms were investigated by feeding on mulberry leaves treated with low concentration of insecticide. Key physiological indicators, including body weight and cocooning rate, were compared between the control and treatment groups. Pathological changes in the silk gland were examined using hematoxylin-eosin staining to assess the impact of novaluron exposure on larval growth and development. Furthermore, the expression levels of genes related to silk protein synthesis, signaling pathways, and major detoxification enzymes were also determined and analyzed. Corresponding changes in detoxification enzyme activities were also measured. This work explored the damage to silk glands and associated transcriptional changes, providing a theoretical basis for understanding the toxic mechanisms of novaluron exposure in silkworms.

## Materials and methods

### Insect strains

The silkworm strain used in this study was P50, which was maintained in the Sericultural Scientific Research Center, Chinese Academy of Agricultural Sciences (latitude: 119.37; longitude: 32.11). The larvae were reared at 28 ± 1 °C with 85% relative humidity (RH) for the first to third instar, while the fourth and fifth instar were maintained at 25 ± 1 °C and 70% RH under ventilated conditions.

### Chemicals

Novaluron was purchased from ADAMA Makhteshim Ltd (Beijing, China). Reagents for total RNA extraction and reverse transcription were obtained from Nanjing Vazyme Biotech Co., Ltd (Nanjing, China). All chemicals for RT-PCR and quantitative real-time PCR (qRT-PCR) were supplied by Beijing TransGen Biotech Co., Ltd (Beijing, China).

### Experimental Material Processing

Silkworm larvae were randomly divided into two groups: a control group and a treatment group. Each group consisted of three replicates, with 90 individuals per replicate. Within each replicate, 30 larvae were allocated for investigating economic indices, 30 were reserved for anatomical dissection, and 30 were designated for enzyme activity assays. Mulberry leaves were treated with a 0.001 mg/L solution of novaluron and air-dried naturally^23^. The treated leaves were provided to the larvae for a single feeding event on the second day of fifth instar. For all other feeding times, the larvae were supplied with fresh mulberry leaves treated with ddH₂O. At 24 h, 48 h and 72 h after exposure, surviving individuals were randomly selected for dissection. The silk glands were then excised, rinsed with ice-cold PBS buffer, and immediately stored at −80 °C. Three independent biological replicates were established for each time point and analysis.

### Histological examination

Silk gland tissues were collected from both the control and treatment groups, fixed in 4% glutaraldehyde for 48 h, and embedded in paraffin. The embedded tissues were then serially sectioned at a thickness of 4 μm, mounted on glass slides, and subjected to standard hematoxylin-eosin staining. Finally, the stained sections were observed under a light microscope.

### Cocoon Quality Assessment

On the sixth day after cocoon formation, the number of cocoons, the number of larvae that failed to cocooning, and the number of dead larvae were recorded and counted. The cocooning rate was subsequently calculated. The cocooning rate is the ratio of cocoon shell weight to cocoon weight. The dead worm cocoon rate is the ratio of number of dead larvae in cocoon to the number of cocoons.

### Total RNA Extraction and Reverse Transcription

Total RNA was extracted using FreeZol Reagent (Vazyme), followed by reverse transcription with the HiScript III 1 st Strand cDNA Synthesis Kit (+ gDNA wiper).

### Quantitative Real-Time PCR (qRT-PCR)

The qRT-PCR primers were designed using Premier 6.0 software, and the sequences are listed in Table S1. qRT-PCR was performed by using the ChamQ Blue Universal SYBR qPCR Master Mix kit. Each sample was run in three technical replicates, and the data are presented as the mean ± SEM (Standard Error of the Mean) of these replicates. The relative transcription levels of the target genes were calculated using the 2^–ΔΔCt^ method^24^.

### Enzyme Activity Assays

100 mg of silk gland tissues from both the control and novaluron-treated groups were collected at 24 h, 48 h, 72 h, and 96 h after treatment. The tissues were homogenized in 1 mL of cell lysis buffer supplemented with 10 µL of phenylmethylsulfonyl fluoride (PMSF). The protein-containing supernatant was then collected and stored at −80 °C for subsequent analysis. The activities of cytochrome P450 monooxygenases (CYPs), glutathione S-transferase (GST), and carboxylesterase (CarE) were measured using specific assay kits purchased from the Nanjing Jiancheng Bioengineering Institute (Jiangsu, China), strictly following the manufacturer’s protocols. The enzyme concentrations were determined by comparing the optical density (O.D.) values of the samples against the standard curve.

### Statistical Analysis

The results are shown as the mean ± standard error (SE) of three independent measurements. To determine the effect of novaluron exposure on the cocooning of silkworms and expression level changes of genes, one-way ANOVAT was performed by different concentrations and exposure times, for comparisons between groups. Asterisks denote significant differences as compared with the control group, as indicated by ^*^ *p* ≤ 0.05, ^**^ *p* ≤ 0.01 and ^***^ *p* ≤ 0.001.

## Results

### Effects of novaluron exposure on larval growth and development of *Bombyx mori*

**Fig. 1:**
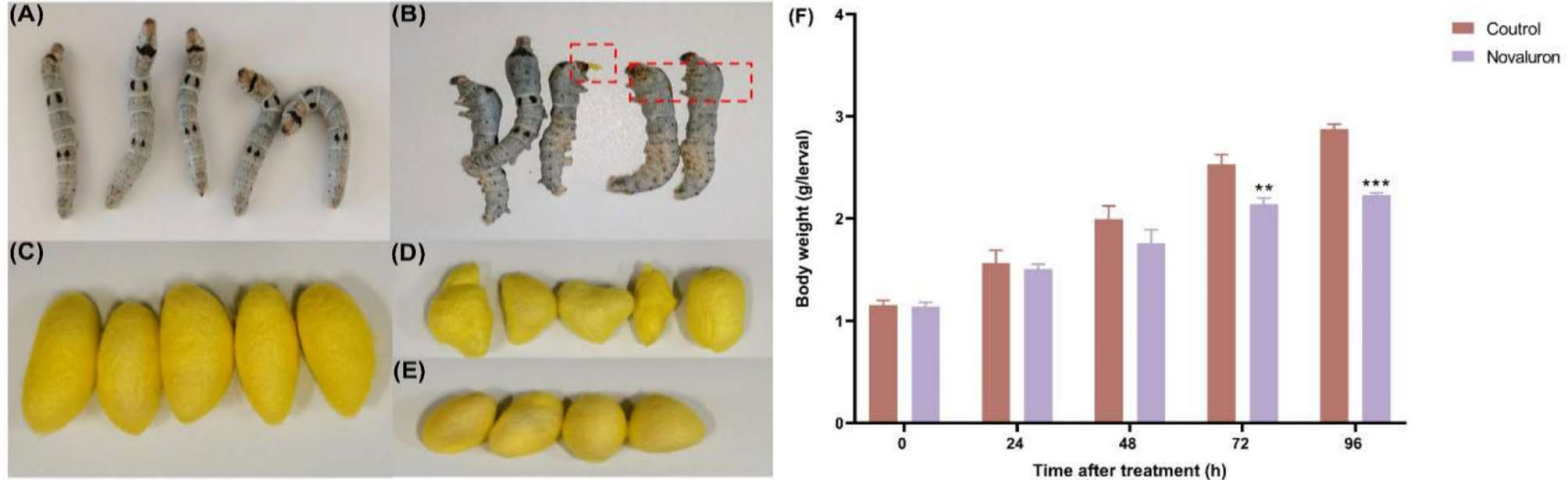
Effect of novaluron exposure on the growth and development of *Bombyx mori* larvae. (A) Control group; (B) Treatment group. Red dotted box indicates symptoms of poisoning; (C) Cocoons from control group; (D-E) Cocoons from treatment group; (F) Body weight changes of 5th instar larvae after novaluron exposure. Asterisks denote significant differences between treatments and controls, as determined using pairwise t-tests (^**^p ≤ 0.01, ^***^p ≤ 0.001). Values represent means ± SEM (*N* = 6).

The growth and development of *Bombyx mori* larvae can be affected by novaluron exposure (Fig. 1). At 24 h after novaluron exposure, the treated silkworms exhibited typical symptoms of intoxication, including reduced feeding, swelling of the head and thorax, body flaccidity, and regurgitation (Fig. 1B). Furthermore, compared to the control group, the treatment group produced cocoons with thinner shells and a higher incidence of malformation (Fig. 1D-E). Concurrently, daily body weight measurements revealed that the treated larvae experienced significant weight reductions of 11.24% and 18.28% at 72 h and 96 h, respectively, relative to the controls (Fig. 1F).

### Effects of novaluron exposure on cocoon quality of *Bombyx mori*

To further investigate the effects of novaluron exposure on the cocooning ability of silkworm, *Bombyx mori*, key cocoon performance indicators were systematically assessed and compared between the control and experimental groups (Table 1). Novaluron exposure significantly reduced the cocooning rate among surviving silkworms and resulted in a markedly higher proportion of dead worm cocoons compared to the control group (*p ≤* 0.01). Additionally, both the whole cocoon weight and the cocoon shell weight were significantly lower in the treated group than those in the control group.

**Table 1:** Effects of novaluron exposure on cocooning and cocoon quality of *Bombyx mori* larvae.

	Control	Novaluron
rate of cocooning (%)	93.33 ± 0.289	41.67 ± 0.577 ^***^
rate of dead worm cocoons (%)	7.11 ± 0.029	28.03 ± 0.056 ^**^
cocoon weight (g/cocoon)	1.09 ± 0.016	0.93 ± 0.066 ^*^
cocoon shell weight (g/cocoon)	0.14 ± 0.012	0.12 ± 0.014
ratio of cocoon shell (%)	12.88 ± 0.080	12.78 ± 0.019

The experiments were repeated six times.

The significance of differences is indicated by ^*^ *p* ≤ 0.05, ^**^ *p* ≤ 0.01, ^***^ *p* ≤ 0.001.

### Histopathological analysis of silk glands after novaluron exposure

**Fig. 2:**
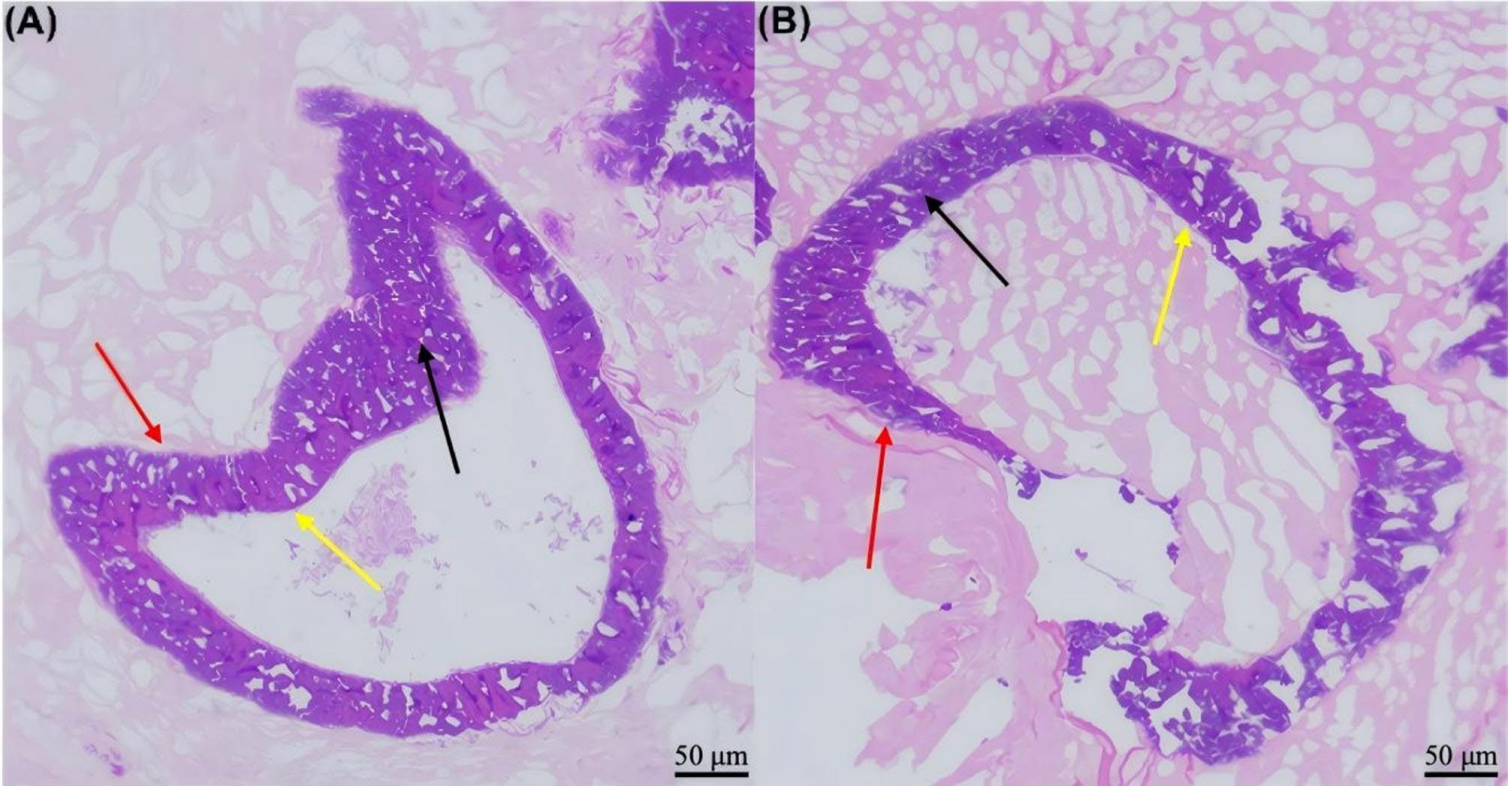
Histopathology of the silk gland tissue in 5th instar larvae of *Bombyx mori*. (A) Control group; (B) Treatment group. Red arrows indicate outer membrane, yellow arrows indicate inner membrane, black arrows indicate glandular cell layer.

Novaluron exposure resulted in significant histopathological alterations in the silk glands of the treated group. The silk glands in the control group displayed an intact structure, a clearly defined lumen, and thin glandular walls (Fig. 2A). In contrast, the silk glands from the novaluron-treated group were severely damaged, characterized by extensive vacuolation and ruptures within the connective tissue layer, indicating cellular necrosis or apoptosis (Fig. 2B).

### Effects of novaluron exposure on genes involved in silk protein synthesis

**Fig. 3:**
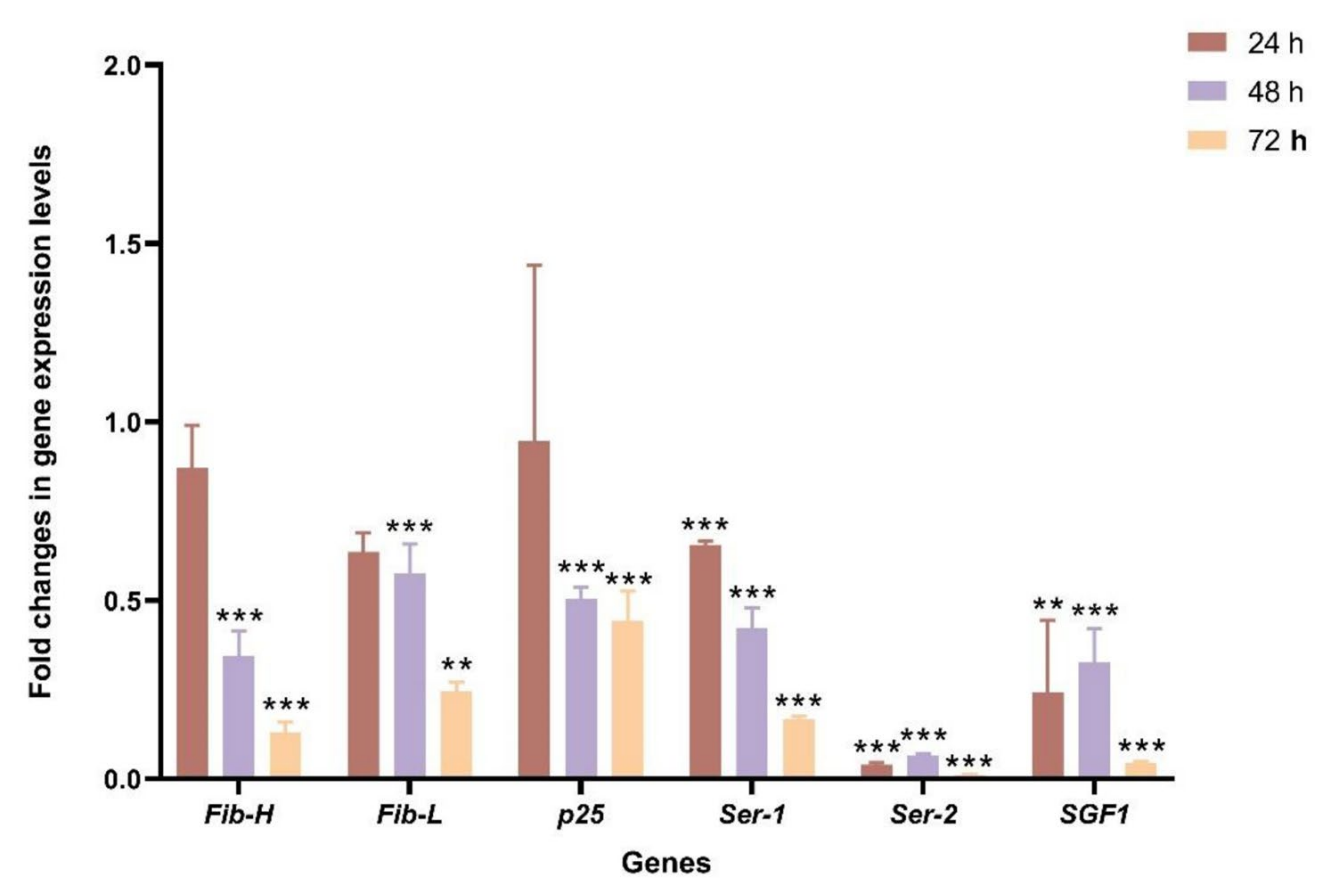
Relative expression levels of domestic silk protein-related genes after novaluron exposure. Fib-H: Fibroin heavy chain, Fib-L: Fibroin light chain, Ser-1: sericin-1, Ser-2: sericin-2, SGF1: silk gland factor 1. Asterisks denote significant differences between treatments and controls, as determined using pairwise t-tests (^**^p ≤ 0.01, ^***^p ≤ 0.001). Values represent means ± SEM (*N* = 6).

The transcriptional levels of silk protein genes, including fibroin components (*Fib-H*,* Fib-L*, and *P25*) and sericin components (*Ser-1*,* Ser-2*, and *SGF1*), were measured using quantitative real-time PCR (qRT-PCR). At 24 h after exposure, the relative expression levels of *Fib-H*, *Fib-L* and *P25* genes were downregulated, with 0.87, 0.636 and 0.946 folds, respectively. Meanwhile, the *Ser-1*, *Ser-2* and *SGF1* genes, which are associated with sericin synthesis, were also significantly downregulated at different time after novaluron exposure (Fig. 3).

### Effects of novaluron exposure on key genes involved in the immune signaling pathways

**Fig. 4:**
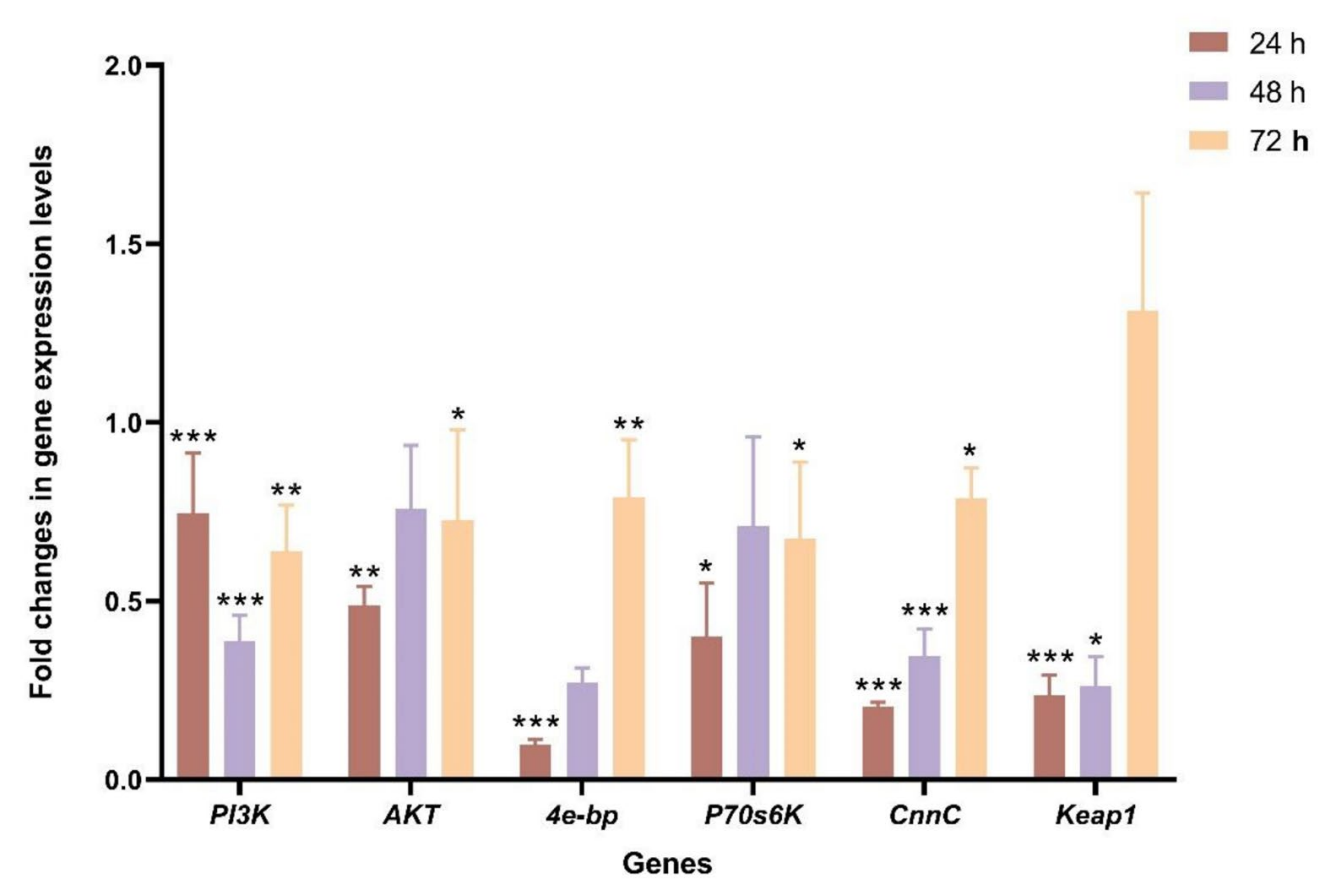
Effect of novaluron exposure on key genes involved in the PI3K/Akt and CncC/Keap1 immune pathways. PI3K: phosphatidylinositol 3-kinase, AKT: protein kinase B, 4e-bp: eif4e-binding protein, P70s6K: P70s6 kinase, CncC: Cap ‘n’ collar isoform C, Keap1: Kelch-like ECH associated protein 1. Asterisks denote significant differences between treatments and controls, as determined using pairwise t-tests (^*^p ≤ 0.05, ^**^p ≤ 0.01, ^***^p ≤ 0.001). Values represent means ± SEM (*N* = 6).

Some key genes involved in the PI3K/Akt and CncC/Keap1 immune signaling pathways were selected and detected in this study, and the results were showed in Fig. 4. The relative expression levels of *phosphatidylinositol 3-kinase* (*Pi3k*), *protein kinase B* (*Akt*), *eif4e-binding protein* (*4e-bp*), *P70s6 kinase* (*P70s6k*), and *Cap ‘n’ collar isoform C* (*CncC*) were significantly downregulated at 24 h, 48 h and 72 h after novaluron treatment compared with control groups. The transcription levels of the *Kelch-like ECH associated protein 1* (*Keap1*) gene at 24 h and 48 h were 0.236 and 0.262 folds over that of the control group, but this gene was upregulated at 72 h after treatment with 1.312 folds (Fig. 4).

### Effects of novaluron exposure on detoxification enzymes in the silk glands of *Bombyx mori*

**Fig. 5:**
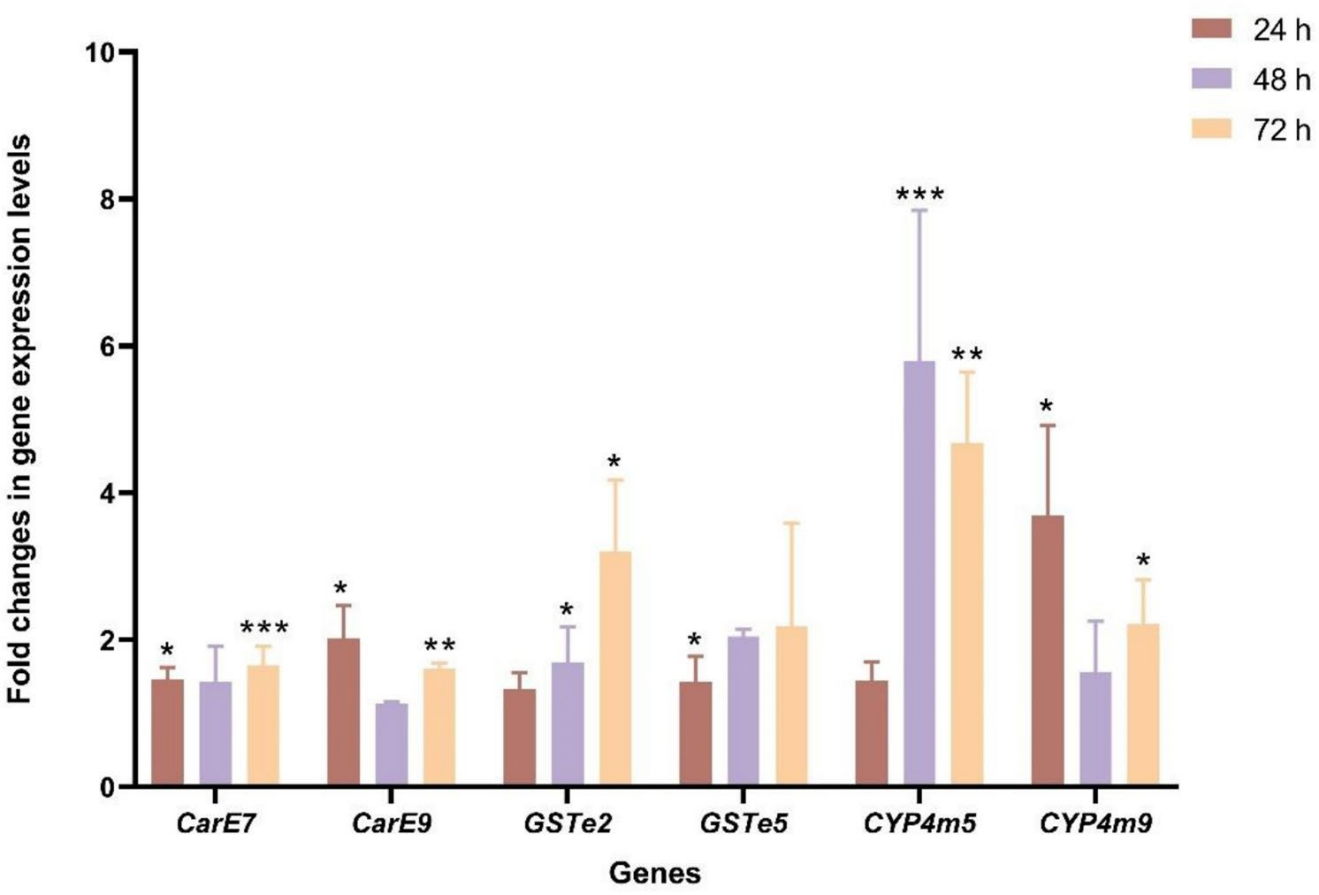
Effect of novaluron exposure on transcription levels of detoxification enzymes genes. CYP(P450): cytochrome monooxygenase; GST: glutathione S-transferase; CarE: carboxylesterase. Asterisks denote significant differences between treatments and controls, as determined using pairwise t-tests (^*^p ≤ 0.05, ^**^p ≤ 0.01, ^***^p ≤ 0.001). Values represent means ± SEM (*N* = 6).

Exposure to novaluron led to the upregulation of all examined detoxification enzyme-related genes (*CarE7*, *CarE9*, *GSTe2*, *GSTe5*, *CYP4m5*, and *CYP4m9*) in the silk gland compared to the control group. Among these, *CYP4m9* showed the most substantial induction at 24 h, with a 3.687 folds increase. Furthermore, the transcriptional level of *CYP4m5* was significantly upregulated by 5.795 folds at 48 h after exposure (Fig. 5).

The activities of three kinds of detoxification enzymes (CYPs, GST, and CarE) were measured at 24 h, 48 h, 72 h, and 96 h after exposure. As shown in Table 2, the results demonstrated that the activities of detoxification enzymes in the novaluron treatment group were significantly higher than those in the control group at all the time points examined.

**Table 2:** Changes of detoxification enzyme activities in the silk gland of *Bombyx mori* at different times after novaluron exposure.

Exposed time(h)	Enzymes activity(U/L)					
	P450 (CYP)		GST		CarE	
	Control	Novaluron	Control	Novaluron	Control	Novaluron
24	15.40 ± 1.21	19.57 ± 0.89 ^**^	171.26 ± 16.04	241.42 ± 14.93	0.59 ± 0.059	0.86 ± 0.054 ^*^
48	20.05 ± 1.13	23.13 ± 1.19	241.14 ± 12.01	292.12 ± 17.01 ^*^	0.56 ± 0.054	0.87 ± 0.076
72	19.44 ± 1.06	24.89 ± 1.15	236.38 ± 15.56	299.82 ± 21.03	0.54 ± 0.037	0.90 ± 0.064 ^**^
96	18.12 ± 1.18	23.70 ± 1.04	202.49 ± 21.69	315.08 ± 20.24	0.73 ± 0.060	0.84 ± 0.062

P450 (CYP): cytochrome monooxygenase; GST: glutathione S-transferase; CarE: carboxylesterase. The experiments were repeated six times. The significance of differences is indicated by ^*^ *p* ≤ 0.05, ^**^ *p* ≤ 0.01.

## Discussion

The structural characteristics of the silkworm silk gland are closely associated with the synthesis and secretion of silk proteins^25^. The fibroin, which is synthesized in posterior silk gland, is transported via intracellular vesicles into the glandular lumen and then conveyed toward the anterior and middle regions^26^. The middle silk gland, which is the thickest part, plays a key role in synthesizing and secreting sericin. The sericin protein not only protects the fibroin from environmental degradation but also imparts flexibility and moisture retention properties to the cocoon silk^27^. In contrast, the anterior silk gland is relatively narrow and does not participate in silk protein synthesis^28^. Novaluron, a benzoylurea insecticide, exerts its effects primarily by inhibiting chitin synthase, accelerating chitinase-mediated degradation, and disrupting ion channels, thereby compromising the integrity of the insect exoskeleton and interfering with chitin synthesis. This action mode ultimately prevents normal molting, leading to developmental arrest and mortality^29^. In this study, silkworms fed mulberry leaves treated with novaluron exhibited severe toxic symptoms. Moreover, the average body weight of larvae was significantly lower than that of the control group. qRT-PCR results showed that the transcription levels of fibroin genes (*Fib-H*, *Fib-L*, and *P25*) were significantly downregulated in the treatment group. Similarly, the expressions of key sericin genes (*Ser-1*,* Ser-2*) and the transcription factor gene *SGF1* were also markedly reduced. In addition, novaluron exposure significantly decreased the cocooning rate and cocoon shell weight, while the mortality rate was higher than that of the control group. These findings indicated that novaluron poisoning adversely affects the growth, development, and physiological metabolism of silkworms. By downregulating silk protein-related gene expression, novaluron exposure suppressed the synthesis of both fibroin and sericin, thereby severely impaired the spinning ability of silkworms and resulted in significant reductions in both cocoon yield and quality.

The PI3K-Akt signaling pathway is known to regulate diverse physiological functions, including cell proliferation and apoptosis. PI3K phosphorylates the substrate PIP2 to generate PIP3, which in turn promotes Akt phosphorylation via PDK1^6^. Akt modulates cellular metabolism by enhancing mRNA translation and protein synthesis, promoting glucose uptake and metabolism, and regulating cell cycle progression. Previous studies have demonstrated that the PI3K-Akt pathway influences cell proliferation, growth, and tissue differentiation in *Drosophila*, and its activation enhances the ability of silkworms to resist exogenous toxic compounds^30,31^. The CncC-Keap1 pathway, which is regulated by PI3K-Akt signaling pathway, contributes to pesticide resistance by controlling the expression of detoxification enzymes and heme oxygenase-1^32^. In this study, novaluron exposure significantly downregulated the expression of key genes in both the PI3K-Akt and CncC-Keap1 pathways in the silk gland, indicating their involvement in the response to novaluron stress. This transcriptional suppression suggested that the antioxidant defense mechanisms and metabolic regulation in silk gland cells may be inhibited under novaluron-induced physiological stress.

Insect detoxification enzymes represent a heterogeneous enzyme system that plays a crucial role in xenobiotic metabolism and protecting insects from oxidative damage^33^. Cytochrome P450 monooxygenases (CYPs) are primarily involved in the metabolism of both endogenous and exogenous compounds through various oxidation reactions, and they also contribute significantly to insect growth and development^34^. Glutathione S-transferases (GSTs) participate in the intracellular transport, detoxification, and metabolism of xenobiotics^35^. Carboxylesterases (CarEs) primarily hydrolyze both endogenous and exogenous compounds containing ester bonds into their corresponding alcohols and acids, thereby metabolizing toxic substances from food or the environment^36^. Previous studies have shown that the CarE family genes can be induced by low concentrations of organophosphates in the midgut of silkworm, which was consistent with increased CarE enzyme activity^37^. Additionally, sublethal doses of insecticides such as deltamethrin and dimethoate significantly induced the expression of certain *CYP450* genes in the Asian gypsy moth (*Lymantria dispar asiatica*), suggesting their important role in insecticide tolerance^38^.

In the present study, the expression of CYP, CarE, and GST genes was upregulated to varying degrees at 24, 48, and 72 h after treatment. The expression levels of *GSTe*2 and *GSTe*5 increased significantly over time, with *CYP4m*5 showing the most pronounced upregulation at 48 h post-exposure. Consistent with these findings, detoxification enzyme activities were also significantly elevated compared to the control group. These results suggested that detoxification enzymes may play a key role in the metabolic detoxification of novaluron in silkworms. Furthermore, the significant increase in enzyme activity provides additional evidence for the activation of the detoxification system in response to novaluron exposure. This indicated that silkworms counteract novaluron-induced stress by upregulating detoxification enzyme expression and enhancing their catalytic activity, thereby initiating metabolic detoxification mechanisms to mitigate the potential toxic effects of novaluron on physiological functions.

## Electronic Supplementary Material

Below is the link to the electronic supplementary material.

Supplementary Material 1

## Data Availability

Most of the analytical data are provided in the article. More original datasets used and/or analyzed during the current study are available from the corresponding author on reasonable request.
