# Supplementary material for: Effects of novaluron exposure on cocooning and transcriptional changes of genes in silk gland of silkworm, Bombyx mori
Source: Sci Rep. 2026 Apr 26;16:19263. doi: 10.1038/s41598-026-50056-6 (PMC13284350; doi:10.1038/s41598-026-50056-6)
Supplement: Supplementary file 1 — Supplementary Material 1 [file 41598_2026_50056_MOESM1_ESM.docx]

**Table S1 Primer sequences for qRT-PCR.**

| **Gene name** | **GenBank**  **Accession No** | **Primer sequence (5′-3′)** | **Length of Product (bp)** |
| --- | --- | --- | --- |
| ***Actin3*** | NM_001126254 | F: CGGCTACTCGTTCACTACC  R: AGCAATTCACACAAGGCAGT | 147 |
| ***Fib-H*** | NM_001113262 | F: ACAAGGTGCAGGAAGTGC  R: TCCTTGATGAGTGCTGTATC | 152 |
| ***Fib-L*** | NM_001044023 | F: CTGGTCAACTCCGATACTC  R: GTCACTGCTGGCTAAGATT | 104 |
| ***P25*** | NM_001145941 | F: CTGCTACTTGGACGATTACA  R: TGGCGTTGAAGTATGGTATT | 134 |
| ***Ser-1*** | NM_001044041 | F: GTTCCAAGAGTCGTGATGA  R: TATCCATAAGTGCTGCTACC | 112 |
| ***Ser-2*** | NM_001172816 | F: TGAAGAGAAGGAGAGGAGTT  R: GTCGTCTGAATCGTATTGC | 187 |
| ***SGF1*** | NM_001043864 | F: CAACCCGACAATCTCATC  R: CACAACGACGGACTCTTC | 119 |
| ***PI3K*** | NM_001127721 | F: GACACTCGTGATGGAACTTTT  R: AACTTATTTGCACCACCTTTC | 92 |
| ***AKT*** | XM_004929860 | F: AAGGTGGTTCTTGAGCCGTGAGA  R: CCGTCGGGAAGTTCGTAAG | 130 |
| ***4e-bp*** | NM_001046944 | F: CTCAAATGCCTGATGTCTATTCC  R: GAAGACGGATTCTTCAGTAGGG | 175 |
| ***P70s6K*** | NM_001130906 | F: TGATTGGTGGAGTCTAGGAGCATTG  R: GCGTGAGGTAGGCAGGTAACATAA | 92 |
| ***CncC*** | XM_038014016 | F: CATGGACGAGTTCAACGAGAG  R: GCGAGCGAGGTTATCTGGT | 147 |
| ***Keap1*** | XM_038018868 | F: ATGACCTGCCTCCGATTAGT  R: TCCAACTTCCAACACGACATC | 173 |
| ***CarE7*** | NM_001114867 | F: GGCTCCTGGTAACAATGG  R: CGGCAATGGTGACTAAGT | 104 |
| ***CarE9*** | NM_001128312 | F: CGAAGATATTACGGATGAAGTC  R: TATGTGTCGTTGATGCTGTA | 189 |
| ***GSTe2*** | NM_001043955 | F: GAAGATGATCTGGTGATTGC  R: GTCGGTTATCGCTCGTATT | 120 |
| ***GSTe5*** | NM_001114992 | F: TCTTGGACAGGATGAATTGA  R: ATGGAAGTAACGCAGCAA | 165 |
| ***CYP4m5*** | NM_001110363 | F: CGAACTTGGAGGACTTGT  R: AGATGTGAATGTGGCAGAA | 162 |
| ***CYP4m9*** | NM_001079666 | F: TAGGCTACGAGACGGATTA  R: CCAGAATGACGCAGAACT | 113 |
